# Severe growth hormone deficiency is rare in surgically-cured acromegalics

**DOI:** 10.1007/s11102-012-0424-6

**Published:** 2012-08-24

**Authors:** Shingo Fujio, Hiroshi Tokimura, Hirofumi Hirano, Ryosuke Hanaya, Fumikatsu Kubo, Shunji Yunoue, Manoj Bohara, Yasuyuki Kinoshita, Atsushi Tominaga, Hiroshi Arimura, Kazunori Arita

**Affiliations:** 1 Department of Neurosurgery, Graduate School of Medical and Dental Sciences, Kagoshima University, 8-35-1, Sakuragaoka, Kagoshima, 890-8520 Japan; 2 Department of Neurosurgery, Graduate School of Biomedical Science, Hiroshima University, 1-2-3, Kasumi, Minami-ku, Hiroshima, 734-8551 Japan; 3 Department of Diabetes and Endocrinology, Graduate School of Medical and Dental Sciences, Kagoshima University, 8-35-1, Sakuragaoka, Kagoshima, 890-8520 Japan

**Keywords:** Acromegaly, Growth hormone deficiency, Surgery, Pituitary function

## Abstract

Growth hormone deficiency (GHD) in surgically-cured acromegalics has been reported to negatively affect their metabolic condition and quality of life (QOL). The incidence of GHD, its causes, and its effects on their physio-psychological condition remain to be examined in detail. We performed a retrospective study to investigate GH secretory function in surgically-cured acromegalics, prognostic factors of GHD, and its impact on QOL. The study population consisted of 72 acromegalics who were determined to be surgically cured according to the Cortina consensus criteria. We recorded the incidence of impaired GH secretory function based on the peak GH level during postoperative insulin tolerance test (ITT) which lowered their nadir blood sugar to under 50 mg/dL. Their QOL was evaluated by SF-36. In surgically-cured acromegalics, the incidence of severe GHD (peak GH during ITT ≦ 3.0 μg/L) was 12.5 % (9/72). The preoperative tumor size was significantly larger in patients with severe GHD than without severe GHD (21.9 ± 9.0 vs. 15.5 ± 7.1 mm, *p* = 0.017). The peak GH levels during postoperative ITT were statistically correlated with the physical but not the mental component summary of the SF-36 score. The incidence of GHD was 12.5 % in our surgically-cured acromegalics. As some QOL aspects are positively related with peak GH levels during postoperative ITT, efforts should be made to preserve pituitary function in acromegalic patients undergoing adenomectomy.

## Introduction

Acromegaly leads to serious metabolic disruptions and intercurrent illnesses, negatively affects the patients’ quality of life (QOL), and increases their mortality rates. Good control of the disease improves their metabolic condition, lessens their morbidity, and raises their life expectancy to that of unaffected individuals [1–3]. Treatments include transsphenoidal surgery under microscopic and/or endoscopic observation, dopamine receptor agonists, somatostatin analogues, GH receptor antagonists, and stereotactic radiation. Due to the introduction of endoscopic surgery and advances in surgical techniques, the surgical cure rate is 60–70 % [4–6].

Growth hormone (GH) affects not only the metabolism and organs but also the mental health of adults and children. And GH deficiency (GHD) disturbs the metabolic condition, lowers QOL, and may lead to premature death due to cardiovascular diseases. Appropriate GH replacement therapy reverses these effects [7, 8]. So the prime treatment goal in patients with acromegaly should be not only the correction of hypersomatotropinemia by eradication of the GH-producing adenoma but also maintenance of the secretory function of GH from the pituitary gland. However, in 40–70 % of acromegalics, GH secretory function has been reported to be impaired postoperatively [9–11]; their metabolic condition is adversely affected and their QOL is decreased [12]. While, Yamada et al. [13] very recently reported a low rate of GHD in patients treated by surgery alone.

We performed a retrospective study on acromegalics treated by selective adenomectomy. They underwent insulin tolerance tests (ITT) 3–12 months after the operation to assess GH secretory function. We also assessed the postoperative secretory function of their other anterior pituitary hormones and QOL. Additionally, their postoperative GH secretory function was compared with that of the patients with clinically nonfunctioning pituitary adenomas treated by total or subtotal adenomectomy.

## Patients and methods

### Subjects

From 1999 through 2011, 136 acromegalics underwent endonasal-transsphenoidal surgery performed or supervised by a senior neurosurgeon (K.A.). All operations were under a microscope and most were aided by endoscopic observation. Surgical cure, judged by Cortina Consensus criteria, i.e. a nadir GH level during the postoperative oral glucose tolerance test (OGTt) <1 μg/L and a normal IGF-1 level, was achieved in 92 of the 136 patients (67.6 %). No patient developed permanent postoperative diabetes insipidus or cranial nerve impairment. We were able to assess postoperative GH secretory function in 89 patients; in 79 this was performed with the ITT and in 10 we used the arginine test because their age and/or persistent hyperglycemia made use of the ITT inadvisable. As the nadir blood glucose level exceeded 50 mg/dL in 7 of the 79 patients who underwent ITT, they were excluded from this study.

The final study population was thus comprised of 72 surgically-cured acromegalics, 25 males and 47 females with age ranging 18–71 years (50.8 ± 12.3, mean ± SD). Their body mass index (BMI) was 17.2–34.9 (24.0 ± 3.4). GH level ranged from 1.55 to 238.6 μg/L (21.12 ± 32.7 μg/L) and IGF-1 level from 170.3 to 1947.6 μg/L (812.0 ± 375.0 μg/L). The tumor size ranged from 5 to 41 mm (16.3 ± 7.6 mm). The mean nadir GH level during postoperative glucose tolerance test was 0.28 ± 0.22 μg/L.

## Methods

For ITT we intravenously injected 0.1–0.15 unit/kg insulin in the morning. The delivered insulin dose was based on the fasting glucose level. Peripheral blood was drawn to measure the level of glucose, GH, adrenocorticotropic hormone (ACTH), and cortisol at 0-, 30-, 60-, and 90-min post-injection. The patient’s condition was closely monitored and if the blood glucose level fell below 25 mg/dL it was adjusted with a 20 % glucose solution.

The patients’ GH secretory status was categorized according to the peak GH level during the test, where peak GH > 6.0 μg/L = normal, 3.0 < peak GH ≦ 6.0 μg/L = slightly impaired, and peak GH ≦ 3.0 μg/L = severe GHD.

In order to assess the function of other pituitary hormones, we also performed luteinizing hormone-releasing hormone (LHRH) and thyrotropin-releasing hormone (TRH) tests and measured target hormones of anterior pituitary hormones on other mornings during a period of 3–6 months after surgery. The determination of hormonal impairment was basically based on a criteria reported by Melmed and Kleinberg [14].

In 43 patients we measured the GH concentration by electro-chemiluminescence-immunoassay (ECL) (Immulite 2000 hGH, Siemens, Erlangen, Germany) using recombinant human GH as the standard. The sensitivity of this assay was 0.1 μg/L. In 29 patients whose GH level was determined by immunoradiometric assay (IRMA) before the introduction of recombinant GH as the standard in 2005, the obtained GH values were converted into ECL GH values using the formula ECL GH value = 0.6 × IRMA GH value. The IGF-1 concentration was measured by IRMA (IGF-1 IRMA “Daiichi”, TFB, Tokyo, Japan) in 27 patients and with the Somatomedin C II Bayer kit (Yuka Medias Co. Ltd., Ibaragi, Japan) in 45 patients. The raw IGF-1 values were assessed according to IGF-1 standard deviation scores (IGF-1-SD-scores) calculated based on the standard IGF-1 values of each gender and age group in the Japanese population [15].

The patients’ QOL was evaluated using SF-36 (ver. 2) questionnaire and presented as relative scores standardized with Japanese general population; the standard value was set at 50.

For comparison we assessed postoperative GH secretory function in 99 patients with clinically nonfunctioning adenomas who underwent total or gross total removal during the same period. They were 49 males and 50 females ranging in age from 14 to 71 years (mean 51.5 ± 12.5 years). Their tumor size ranged from 11 to 52 mm (mean 28.4 ± 8.8 mm).

### Statistical analysis

The Statflex software program (version 6.0) was used for statistical analysis of the results. Depending on the characteristics of the data sets, data were analyzed with Fisher’s exact test, the Mann–Whitney *U* test, Student’s *t* test, or a simple correlation test. Differences of *p* < 0.05 were considered statistically significant.

### Ethical considerations

This retrospective study was approved by the Ethics Committee of Kagoshima University Hospital (reference No. 18-53 and No. 20-144). All investigated data were acquired in a routine fashion and were essential for the proper management of the patients with pituitary disease. To protect patient privacy all data were analyzed under anonymization in a linkable fashion.

## Results

### Prevalence of GH impairment

**Fig. 1:**
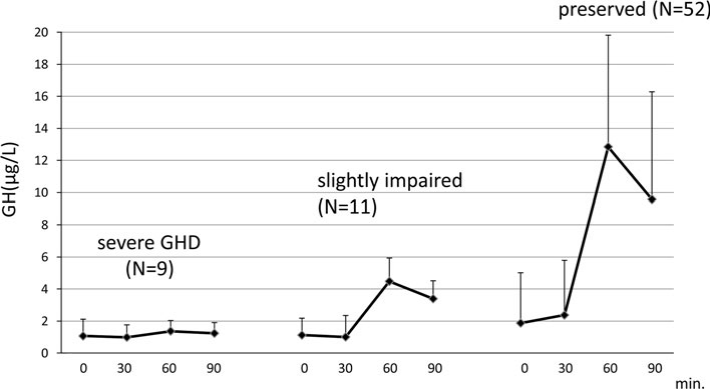
Changes in growth hormone (GH) levels during the postoperative insulin tolerance test (ITT) in 72 surgically cured acromegalics. From *left* to *right*; 9 patients with severe growth hormone deficiency (GHD), 11 with slightly impaired-, and 52 with normal GH secretion. *Bars* indicate the standard deviation (SD)

**Fig. 2:**
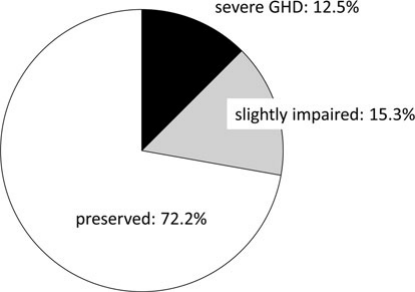
Postoperative status of GH secretory function in 72 surgically cured acromegalics. The incidence of severe GHD was 12.5 %

The incidence of postoperatively impaired GH secretory function was 12.5 % (9/72) in patients with severe GHD (peak GH during ITT ≦ 3 μg/L) and 15.3 % (11/72) in patients with slightly impaired GH secretory function (3.0 μg/L < peak GH ≦ 6.0 μg/L) (Figs. 1, 2). In 52 patients (72.2 %) GH secretory function was preserved; their peak GH exceeded 6 μg/L.

**Fig. 3:**
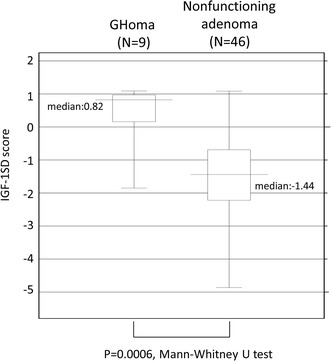
*Box plots* of the IGF-1-SD-scores of severe GHD patients. *Left column* surgically cured acromegalics (n = 9). *Right column* patients with totally or subtotally removed nonfunctioning pituitary adenomas (n = 46). The difference is statistically significant (*p* < 0.001, Mann–Whitney)

**Fig. 4:**
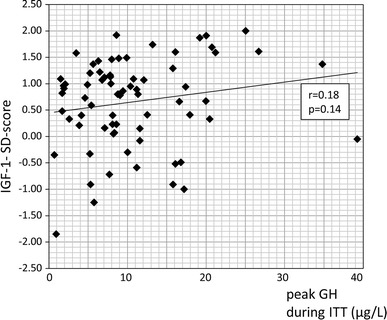
Relationship between the peak GH concentration and the IGF-1-SD-score during postoperative ITT in surgically cure acromegalics

When we set 16 mm as the cut-off, mean of all the tumor sizes, 25.9 % of patients with tumors larger than 16 mm and 4.4 % of patients with tumors smaller than 16 mm had severe GHD (*p* = 0.01, Fisher’s exact test). The median IGF-1-SD-score of 9 patients with severe GHD was 0.82 with mean of 0.38 ± 0.95 (SD) (Fig. 3). IGF-1-SD-score tended to correlate with the peak GH concentration during ITT although the association was not significant (*p* = 0.14) (Fig. 4).

The postoperative incidence of severe GHD in patients with nonfunctioning pituitary adenomas was as high as 46.5 %. The median of IGF-1-SD-score of 46 patients with nonfunctioning pituitary adenomas and postoperative severe GHD was −1.44 with mean of −1.43 ± 1.36 (SD) (Fig. 3).

We judged anterior pituitary hormonal functions as intact when the test results met the following criteria. For ACTH-cortisol; the peak cortisol level was >15 μg/dL. For TSH; normal levels of TSH (0.5–5.0 μIU/mL) and FT4 (>1.1 ng/dL) and the peak TSH level was >2.5 times the basal level. For prolactin; the peak prolactin level was >20 μg/L and 2.5 times the basal level. For gonadotropin for female: menstrual state-appropriate estradiol level and peak LH level was >15 mIU/mL and 2 times the basal level. For gonadotropin for male: normal testosterone level (>2 ng/mL) and peak LH level was >15 mIU/mL or 2 times the basal level. The impairment rate was 8.3 % for the ACTH-cortisol axis, 9.7 % for TSH, 23.6 % for gonadotropin, 6.9 % for prolactin, respectively.

Clinical and endocrinologic data on patients with and without severe GHD are presented in Table 1. The tumor size was significantly larger in the severe GHD group (peak GH ≦ 3 μg/L) than the non-severe GHD group (21.9 ± 9.0 vs. 15.5 ± 7.1 mm, *p* = 0.017, Mann–Whitney *U* test). Other clinical features including the patients’ age, sex, and their pre- and post-operative GH and IGF-1 level did not affect the tendency for severe GHD.

**Table 1:** Clinical and endocrinologic status in 9 patients with severe growth hormone deficiency (GHD) and 63 without severe GHD

	Severe GHD	Non-severe GHD	*p* value
Number of cases	9	63	
Preoperative characteristics			
Ages(years)	54.8 ± 9.3	50.3 ± 12.3	0.38^a^
Sex(male/female)	1/8	24/39	0.15^b^
BMI	23.2 ± 2.6	24.1 ± 3.5	0.48^a^
Preoperative GH levels	48.6 ± 77.2	17.2 ± 18.2	0.59^a^
Preoperative IGF-1 levels	826.9 ± 354.8	809.9 ± 380.5	0.78^a^
IGF-1-SD-score	7.76 ± 2.4	7.44 ± 2.74	0.62^a^
Tumor sizes	21.9 ± 9.0	15.5 ± 7.1	0.017^a^
Follows-up results			
Nadir GH levels on 75 g OGTt	0.32 ± 0.26	0.27 ± 0.22	0.63^a^
IGF-1 levels	160.9 ± 47.0	218.9 ± 87.4	0.055^a^
IGF-1-SD-score	0.38 ± 0.95	0.70 ± 0.81	0.37^a^

*SDS* Standard deviation score, *SD* standard deviation

^a^Mann–Whitney *U* test

^b^Fisher’s exact test

**Fig. 5:**
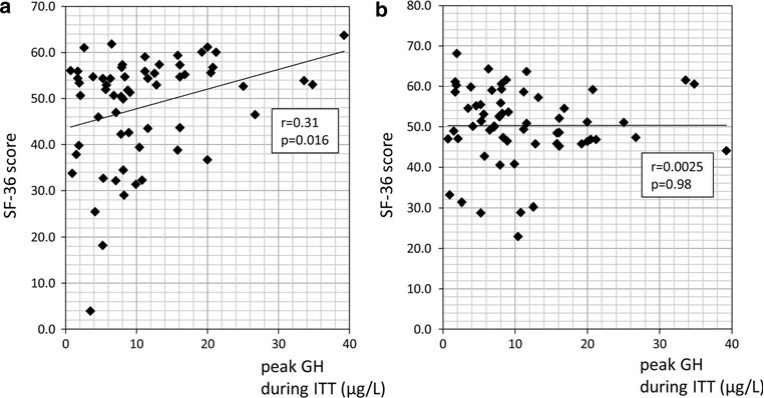
Relationship between the peak GH concentration and the physical (**a**) and mental-component scores (**b**) of SF-36 in surgically-cured acromegalics. The physical component scores correlated positively with the peak GH concentration during ITT (r = 0.31, *p* = 0.016, simple correlation test)

We did not find a difference in the SF-36 scores between patients with (n = 9) and without (n = 63) severe GHD. On the other hand, the peak GH level correlated statistically with the physical component summary of SF-36 (r = 0.31, *p* = 0.016, Fig. 5) but not with its mental component.

## Discussion

It has been shown that 30–70 % of acromegalics develop GHD after radiotherapy [9, 16–18]. Although there was a report that surgical treatment without radiation rarely caused GHD[16], high incidences, more than 50 %, of GHD in surgically cured acromegalics were reported [9, 12]. The incidence of severe GHD in surgically-cured Japanese acromegalics was 9.1 %; their postoperative GH secretory function was evaluated by GHRP-2 test [13]. The rate of severe GHD was also low, 12.5 %, in our patients with surgically cured acromegalics whose GH secretory function was determined by ITT. Differences in the reported rates of severe GHD may be attributable to difference in the study population, the tumor size, the methods used to evaluate GH secretory function, the interval after completion of treatments, the metabolic background, and the manipulation of the pituitary gland during surgery.

In a series reported by Ronchi et al. [9] in 2009, 18 of 33 (54.5 %) patients treated by surgery alone manifested severe GHD. Among 45 cured acromegalics studied by Wexler et al. [12], including 26 who underwent postoperative radiation therapy, 26 (57.8 %) were GH deficient. Considering these results were reported from large volume centers, the number of patients selected for the assessment of post-treatment GH secretory function may be a small part of actual number of acromegalics treated at the institutions.

On the other hand, Yamada et al. [13] included all operated patients who were regularly followed between 2009 and 2010 and our study population was comprised of almost all surgically-cured patients who underwent ITT between 1999 and 2011. Therefore, the low incidence of GHD in the series of Yamada et al. and ours may better reflect the actual status of GH secretory function in surgically-cured acromegalics.

In our series, as in patients reported by Ronchi et al. [9], the only prognostic factor for severe GHD was the preoperative size of the adenomas. This suggests that the impairment of GH secretory function began before surgery due to the compression of the pituitary gland and/or pituitary stalk by large tumors. The incidence of severe GHD was 46.5 % in patients who underwent total or subtotal removal of nonfunctioning adenomas in our series. According to Greenman et al. [19], 17 and 68 % of operated patients with somatotropinomas and nonfunctioning adenomas, respectively, manifested pituitary deficiency. As the tumor size was a prognostic factor for GHD and as there was a big difference between somatotropinomas and nonfunctioning pituitary adenomas (mean size 16.3 vs. 28.4 mm), the incidence of severe GHD in surgically-cured acromegalics can be expected to be much lower than that in patients with nonfunctioning pituitary adenomas, which actually was 12.5 and 46.5 %, respectively in our series.

The optimal timing for the postoperative evaluation and the ideal time to start hormone replacement therapy in patients with GHD remain to be identified. We performed ITT 189.9 ± 188.1 (mean ± SD) days after surgery. In some studies pituitary function recovered over time [20, 21]. Ronchi et al. [9] reported a negative correlation between peak GH and the duration of follow-up after the first treatment. Their observation must be confirmed by follow-up studies of a fixed group of patients.

The low incidence of GHD in Japanese patients may be attributable to a low obesity rate; the body mass index (BMI) in our patients was 24.0 ± 3.4 (SD), lower than that in non-Japanese reported by others [9, 22–24]. In obese patients GH release is depressed, as are 24-h GH secretion, and the responsiveness to provocation tests [25–28].

The low incidence of GHD may also be related to the growth direction of somatotropinomas; they very often grow inferiorly without dislocation or compression of the pituitary gland [29, 30]. Thus, preservation of GH secretory function may be attributable to the anatomical conservation of the pituitary gland in patients with these tumors.

Growth hormone deficiency has adverse effects on the QOL [7, 8, 31–33] which is the case in cured acromegalics [12, 34]. In our series, the peak GH value during ITT correlated with the physical but not the mental component of SF-36. Strong correlation of QOL to peak GH during GH provocation test was also reported by Wexler et al. [12]. But, we found no difference in the SF-36 scores of 9 patients with severe GHD and 63 without severe GHD, reasons remain to be elucidated. We posit that the incorrect belief of being perfectly healthy due to successful surgery in some surgically-cured acromegalics may have brought about excessively high postoperative QOL scores and cancelled the difference between two groups. Actually, in our 26 acromegalic patients whose pre- and post-operative QOL were evaluated, the mental components summary of SF-36 were 46.7 ± 12.6 and 51.4 ± 8.9, respectively; the standard value was set at 50 (*p* = 0.049).

Another factor to explain the lack of difference of QOL between severe GHD and non-severe GHD groups may be the relatively well-maintained IGF-1 level in acromegalics manifesting severe GHD. The mean IGF-1-SD-score in 9 cured acromegalics with severe GHD in our series was significantly higher than in cured patients with nonfunctioning adenomas who had severe GHD (0.38 ± 0.95 vs. −1.43 ± 1.36, Fig. 3). A similar observation was made by Ronchi et al. [9]; cured acromegalics with GHD had a higher IGF-1-SD-score than did GHD patients treated for other pituitary diseases. Thus, mild GH excess and/or GH dysregulation may exist in surgically-cured acromegalics and may account for their relatively maintained IGF-1 level in the presence of severe GHD and may contribute to the maintenance of some QOL components.

In conclusion, we report that a small proportion of surgically-cured acromegalics fulfilled the criteria for severe GHD and that the physical aspect of their SF-36 scores correlated positively with the peak GH level in the ITT. The preoperative tumor size was a predictive factor for postoperative severe GHD. Considering the reported longitudinal changes in GH secretory function in cured acromegalics, future studies should include serial examination during long term follow-up.
